# Exploring the impact of family background and English as a foreign language teachers’ feedback literacy on undergraduates’ critical thinking styles

**DOI:** 10.3389/fpsyg.2025.1721487

**Published:** 2026-01-16

**Authors:** Wenjuan Ma, Yadan Li, Xiaoshu Xu, Yi Huang, Yixin Pan, Yiyin Li

**Affiliations:** 1 School of Education, Baoshan University, Baoshan, China; 2 Ministry of Education (MOE) Key Laboratory of Modern Teaching Technology, Shaanxi Normal University, Xi’an, China; 3 School of Foreign Studies, Wenzhou University, Wenzhou, China; 4 School of Foreign Languages, Xinyang Normal University, Xinyang, China; 5 Human Resources Department, Wenzhou Polytechnic, Wenzhou, China; 6 School of Education, City University of Macau, Macau, China

**Keywords:** critical thinking style, teacher feedback literacy, family background, EFL teacher, family education

## Abstract

**Introduction:**

The present study investigated the relationship between family background and English as a Foreign Language (EFL) teachers’ feedback literacy on the critical thinking styles of undergraduates in Chinese universities.

**Methods:**

We collected data from 1,454 undergraduates using the validated measures of the validated measures of the University of Florida Critical Thinking Inventory (UFCTI) and the EFL Teacher Feedback Literacy Scale. In addition, they reported on family background indicators, including parental education level, family income and family educational capital. We conducted chi-square tests and multivariate binary logistic regression analyses with two types of critical thinking styles as dependent variables.

**Results:**

The study revealed the following findings: (1) Students from more advantaged families were more likely to be Engagers, whereas students from less advantaged backgrounds were more likely to be Seekers. (2) Higher EFL teacher feedback literacy was associated with a greater likelihood of the Seeker style. (3) There was a significant interaction between family educational capital and EFL teacher feedback literacy; specifically, the effect of EFL teacher feedback literacy on critical thinking styles in students from families with lower educational capital was enhanced.

**Discussion:**

The results of this study indicate that critical thinking styles are contextually sensitive cognitive approaches, which impacted by both an individual’s family background and the ecology of feedback received in EFL classes. Besides, educators and policymakers should design feedback and support systems that recognize style diversity while mitigating inequalities linked to family background.

## 1 Introduction

In an era of rapid globalization and technological advancement, critical thinking has emerged as a fundamental cognitive competency essential for academic success, career readiness, and lifelong learning (Geng et al., 2024; Xu et al., 2023). It encompasses a set of cognitive skills (e.g., analysis, evaluation, inference) and dispositional tendencies (e.g., open-mindedness, truth-seeking) that together support purposeful, self-regulatory judgment in addressing questions, forming reasoned judgments, making decisions and solving problems (Andreucci-Annunziata et al., 2023; Hyytinen et al., 2025). As critical thinking becomes a core competency in higher education, understanding how students develop distinct critical thinking styles has significant implications for pedagogical practices and educational policy.

Critical thinking styles defined as a learner’s characteristic approach to interpreting information, engaging with problems and generating solutions (Fan and See, 2022; Xu et al., 2025). Importantly, critical thinking styles do not index higher or lower levels of cognitive ability; instead, they reflect individuals’ preferred modes of engagement with complex tasks. At one pole sit engagement-oriented thinkers, who exhibit dialogic involvement, collaborative reasoning and a willingness to challenge assumptions. At the other pole are information-seeking thinkers, who rely on systematic information gathering, cautious evaluation and reflective deliberation before forming conclusions (Lu et al., 2021; Juliyanti and Syahfitri, 2024).

Despite advances in measurement and validation of these styles (Baker et al., 2021), much less is known about the antecedent conditions that shape their development. Understanding these antecedents is essential because thinking styles do not emerge in a vacuum; rather, they are embedded within learners’ sociocultural ecologies. Two microsystems are particularly influential. The first is the family environment, which provides early cognitive modeling and educational resources that shape learners’ epistemic habits and reasoning preferences (Tan, 2024; Wan, 2022). The second is the classroom feedback environment, especially teacher feedback literacy, which governs how feedback is designed, communicated and taken up by students (Carless and Winstone, 2023; Orhan, 2023).

Theoretically, these two systems may not only exert independent effects but also interact to configure students’ thinking styles in significant ways. However, the field still lacks an integrated understanding of how family background and EFL feedback literacy jointly affect undergraduates’ critical thinking styles, or whether teacher feedback literacy can compensate for, or inadvertently amplify, family-based disparities in thinking patterns.

Against this background, the present study investigates how the critical thinking styles of undergraduates are affected by: (a) their family background; (b) the feedback literacy of their EFL teachers; and (c) the interaction between these two microsystem factors. By examining these relationships within a large sample of Chinese EFL undergraduates, the study aims to further the theoretical understanding of critical thinking style development and inform pedagogical approaches that support cognitively diverse learners.

## 2 Literature review

### 2.1 Family background and its impact on critical thinking styles

It is widely acknowledged that family background exerts a significant influence on students’ cognitive and academic development. This influence is characterized by the shaping of cognitive and metacognitive skills (e.g., executive functions, metacognitive knowledge), academic engagement, and academic self-efficacy (González et al., 2024; Holzer et al., 2025). The concept of family background is typically operationalized by socioeconomic status, parental education level, and family educational capital. These factors influence students’ access to intellectual stimulation, educational resources, and opportunities for reflective dialogue.

From a cultural reproduction perspective, following Bourdieu’s theorization of cultural capital, recent research conceptualizes parental education, family income and home learning practices as intergenerationally transmitted cultural resources that students can mobilize in educational contexts (Djekourmane et al., 2025; de Moll et al., 2024; Nunes and Andrade, 2024). It has been demonstrated that students from higher socio-economic groups and those from families with greater educational attainment are better equipped to meet the demands of academic discourse (Helin et al., 2023; Liu et al., 2022). This is due to the fact that they possess competencies that closely align with institutional expectations, thereby facilitating a smoother transition into the academic environment. Families with abundant cultural and educational resources have been observed to engage their children in intellectually stimulating conversations, encourage exploration, and support independent reasoning (Jin et al., 2024; Yu et al., 2022). Such an environment is more likely to foster an engagement-based critical thinking style, characterized by active engagement, collaborative problem-solving and confident argumentation. Conversely, students from families with limited educational backgrounds or economic resources often have restricted access to such cultural resources. This leads them to rely more heavily on external information sources and favor structured learning approaches, making them more likely to develop an information-seeking style of critical thinking.

From a contemporary social cognitive perspective, individuals acquire and refine cognitive and behavioral patterns through observing significant others, internalizing modeled behaviors, and evaluating their own efficacy in similar tasks (Chen and Hu, 2021; Song and Zhao, 2025). In families with higher socioeconomic resources and better-educated parents, children are more likely to experience cognitively rich interactions, such as analytic conversations about schoolwork, evaluative reasoning and joint problem solving, which in turn strengthen their academic self-efficacy and foster more proactive approaches to learning (Paulus et al., 2021; Shengyao et al., 2024). Conversely, in less advantaged households, the absence of such modeling restricts opportunities for cognitive apprenticeship, compelling students to adopt compensatory, information-seeking strategies to construct their understanding from external structures and feedback.

Thus, family background may influence undergraduates’ thinking style preferences on intergenerational transmission of resources, habits and modeling.

Accordingly, the following hypothesis is proposed: H1: Family background significantly influences undergraduates’ critical thinking styles. Specifically, students from more advantaged family backgrounds are more likely to adopt an engagement-based style, whereas those from less advantaged family backgrounds tend toward an information-seeking style.

### 2.2 EFL teachers’ feedback literacy and its impact on critical thinking styles

In recent years, research on assessment and feedback in higher education has increasingly shifted from viewing feedback as a one-way transmission of corrective comments to conceptualizing it as a dialogic, learner-centred process that supports self-regulation and deeper learning (Carless, 2022). Within this paradigm, teacher feedback literacy has been proposed as a key professional capacity. It is typically defined as teachers’ knowledge, expertise and dispositions to design feedback processes in ways that enable student uptake and foster the development of student feedback literacy (Carless and Winstone, 2023).

In the context of EFL teaching, a feedback-literate teacher demonstrates four interrelated capacities: (1) Designing feedback – planning the timing, focus, resource allocation and staged tasks of feedback; (2) Learning feedback – actively engaging in professional development, classroom observation and peer-sharing to build feedback knowledge; (3) Building feedback consensus – cultivating students’ awareness of their role in the feedback process and guiding their effective participation; (4) Modeling feedback– exemplifying feedback use by drawing on scoring criteria or exemplar work in order to develop students’ feedback literacy and capacity to act on standardized feedback (Xu and Zhang, 2023).

In Chinese university EFL classrooms, the ways in which feedback literacy is enacted are shaped by several contextual characteristics. First, large class sizes and heavy teaching loads make it challenging to provide highly individualized, interactive feedback to every student on a regular basis (Lei and Lei, 2025). Second, English teaching remains strongly influenced by exam-oriented educational traditions, where success is often defined through high-stakes tests and standardized writing or reading tasks (Zhao, 2023). In such settings, both teachers and students place a high value on accuracy-focused corrective feedback and explicit guidance about how to meet assessment standards. Studies of Chinese EFL learners’ feedback preferences consistently show favorable attitudes toward written corrective feedback and a strong preference for extended comments on both content and grammar, as well as clear suggestions for revision (Cheng and Liu, 2022). Classroom observations similarly suggest that oral and written feedback in Chinese university EFL courses tends to concentrate on error correction and provision of linguistic and task-related information, with relatively fewer opportunities for extended dialogic negotiation of ideas (Lwin and Yang, 2021; Xie and Liu, 2024).

Against this backdrop, feedback-literate EFL teachers in China are distinguished not only by the four capacities defined earlier (designing feedback; learning feedback; building feedback consensus; modeling feedback), but also by their ability to operationalize these capacities in high-pressure settings marked by large class sizes, heavy teaching loads and high stakes exam-oriented cultures. For example, they make expectations explicit through rubrics, model texts and annotated exemplars; provide detailed written and oral comments that locate errors, explain underlying rationale and offer concrete revisions; organize staged feedback cycles with clear criteria and checkpoints; and leverage efficient formats or peer-review mechanisms to maintain sustainable, information-rich and criterion-referenced feedback delivery under practical constraints. Thus these teachers translate their feedback-literacy into structured, information-rich, criterion-referenced feedback processes, an enactment that sets the stage for exploring how feedback literacy influence students’ critical thinking styles.

Specifically, such practices align closely with the characteristics of the information-seeking thinking style, in which students focus on gathering, analyzing and applying external input before forming independent arguments. Moreover, in large-class, exam-oriented Chinese university settings, those feedback processes may reduce opportunities for open exchange and co-constructive reasoning, thus tilting the balance further toward an information-seeking style. Therefore, within this educational ecology, it is plausible to expect that higher levels of teacher feedback literacy will be associated with a greater likelihood of students adopting an information-seeking rather than an engagement-oriented critical thinking style.

Building on this reasoning, the present study proposes the following hypothesis: H2. In the Chinese university EFL context, higher levels of teachers’ feedback literacy are associated with an increased likelihood that undergraduates adopt an information-seeking critical thinking style rather than an engagement-oriented style, reflecting.

### 2.3 The interplay between family background and EFL teachers’ feedback literacy in relation to critical thinking styles

The development of undergraduates’ critical thinking styles does not occur within isolated systems but at the intersection of multiple proximal environments. Bronfenbrenner’s ecological systems theory emphasizes that learner development is shaped by interactions between microsystems, most notably the family and the school, and by the quality of the mesosystem linking them (Chen et al., 2025). In this view, family background provides early socialization into particular ways of reasoning, communicating and approaching problems, while the classroom, through teaching, assessment and feedback, either consolidates or redirects these trajectories. Critical thinking styles, conceptualized as relatively stable preferences for engagement-oriented versus information-seeking approaches, can thus be regarded as emergent properties of this home–school interface.

Building on this framework, the preceding sections suggest two complementary forces. First, students from more advantaged family backgrounds, characterized by higher parental education, income and educational capital, are more likely to be socialized into dialogic, exploratory and argumentation-rich practices, predisposing them toward an engagement-based critical thinking style (Lurie et al., 2021; Von Stumm et al., 2020). In contrast, students from less advantaged homes, with fewer intellectual resources and less exposure to cognitively demanding modeling, are more likely to rely on structured materials, external guidance and rule-based processing, aligning with an information-seeking style that emphasizes careful information gathering and cautious evaluation.

Second, within Chinese university EFL classrooms, high levels of teacher feedback literacy tend, given large classes and exam-oriented constraints, to manifest primarily as structured, information-rich, criterion-referenced feedback rather than as sustained dialogic interaction for all students. As argued before, such feedback ecologies naturally support and cultivate an information-seeking style by encouraging students to systematically consult comments, rubrics and model texts, analyze gaps, and revise cautiously. Under these conditions, teacher feedback literacy is more likely to refine and strengthen an information-seeking critical thinking style than to universally transform learners into highly engagement-oriented thinkers.

The interaction between these two forces can be conceptualized through resource substitution theory (Burger and Brack, 2025; Stienstra et al., 2021). This framework posits that when individuals lack certain resources (e.g., family socioeconomic or educational capital), other resources (e.g., schooling, teacher support) become disproportionately important in compensating for those deficits. Applied to the present context, EFL teachers’ feedback literacy functions as an institutional resource that may particularly influence the critical thinking styles of students from less advantaged families, who cannot draw on rich home-based cognitive scaffolding. For these learners, structured, information-dense feedback can substitute for limited family capital by teaching them how to use external information strategically, thereby fostering a more systematic information-seeking style. By contrast, students from more privileged backgrounds may rely more heavily on existing reasoning habits. Consequently, their engagement-oriented style may be less responsive to variations in EFL teacher feedback literacy. Recent research offers preliminary support for this compensatory view. Quinton (2022) shows that teacher effects are stronger than school effects, and that differential teacher perceptions, support and instructional style can significantly impact students from disadvantaged homes.

Taken together, the present study advances the following hypothesis: H3. Family background and EFL teachers’ feedback literacy interact to influence undergraduates’ critical thinking styles. In particular, higher levels of teacher feedback literacy are expected to exert a stronger effect on students from less advantaged family backgrounds, increasing their likelihood of adopting a feedback-driven information-seeking style and thereby partially compensating for limited family educational capital.

## 3 Current research

**Figure 1:**
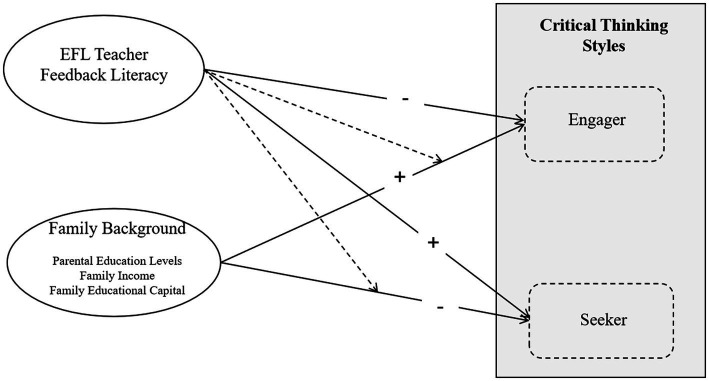
Conceptual model of the associations between family background, EFL teachers’ feedback literacy, and critical thinking styles. Expected effects are + positive, − negative.

Building upon the aforementioned theoretical frameworks and addressing gaps in the existing literature, the present study aims to investigate the effects of family background and EFL teachers’ feedback literacy on undergraduates’ critical thinking styles. Figure 1 demonstrates the Conceptual model. The particular assumptions are as follows:

*H1*: Family background significantly influences undergraduates’ critical thinking styles. Students from more advantaged backgrounds are more likely to adopt an engagement-based style, whereas those from less advantaged backgrounds are more likely to adopt an information-seeking style.

*H2*: Higher levels of EFL teachers’ feedback literacy are associated with a greater likelihood of students adopting an information-seeking critical thinking style.

*H3*: Family background and EFL teachers’ feedback literacy will interact to influence undergraduates’ critical thinking styles. Specifically, the influence of EFL teachers’ feedback literacy is hypothesized to be more pronounced among students from less advantaged family backgrounds.

Conceptual model of the associations between family background, EFL teachers’ feedback literacy, and critical thinking styles. Note. Expected effects are + positive, − negative.

## 4 Methods

### 4.1 Participants and procedure

The study proposal submission form was submitted to and approved by the academic committee of the corresponding author’s university in January 2024 (No. 20240115). Participants filled out the questionnaire via the Wenjuanxing platform between February and April 2024. Participation in the study was completely voluntary. Upon accessing the survey, participants were directed to a page that provided a brief overview of the study, along with information regarding their rights to privacy and anonymity. They consented to take part by clicking ‘agree to continue.

Convenience sampling and snowball sampling procedures were both used to collect participants for the study. Convenience sampling entailed recruiting participants who happened to be available and willing to take part. This technique proved particularly effective in achieving a large sample of respondents in a short space of time. To further increase the sample and obtain a wider range of participants, snowball sampling was used. Participants were asked to refer additional potential respondents, and the sample expanded through a chain of acquaintances.

This mixed design of convenience sampling and snowball sampling allowed the gathering of information from a large and diverse pool of participants to obtain a wide variety of opinions regarding the impact of the family background and the feedback literacy of the EFL teacher on the Critical thinking style of the students. Although these types of non-probability samples are useful for quickly gathering enormous samples, they result in sample selection bias. To address this issue, we incorporated variables like gender and major into our data analysis as control variables. Additionally, we performed a multivariate regression analysis to assess the stability of our findings.

A total of 1,454 students participated in the survey, with the age concentration of 18-25-year-olds (96%); there were 529 males (36.4%) and 925 females (63.6%); Their reported academic majors were as follows: 764 in social science (52.5%), 430 in natural science (39.6%) and 260 in engineering (17.9%); The distribution of the grades they attended are1,078 freshmen, 74.1%, 176 sophomores, 12.1%, 143 juniors, 9.8%, and 57 seniors, 3.9%; About English level: 1,193 have not yet passed the CET-4, 82%, 173 have passed the CET-4, 11.9%, 80 have passed the CET-6 or TEM-4, 5.5% and 8 have passed the TEM-8, 0.6%.

### 4.2 Measures

#### 4.2.1 The university of Florida critical thinking inventory (UFCTI)

The UFCTI was used to assess participants’ critical thinking styles, focusing on the dimensions of seeking information and engagement (Lamm and Irani, 2011). This 20-item inventory employs a five-point Likert scale, with 13 items measuring information-seeking behavior and 7 assessing engagement. Scores were computed for each dimension, with engagement scores adjusted using a constant (1.866) for comparability (Leal et al., 2017; Putnam et al., 2017). Participants with scores of 79 and above were labeled as information seekers, and those with scores 78 and below were defined as engagers (Lamm and Irani, 2011). To reach the Chinese learners with lower English proficiency levels, the UFCTI was also translated into Chinese (Baker et al., 2021). The UFCTI in the current study had very good reliability with Cronbach’ s alphas for engagement (0.95), information-seeking (0.96), and overall (0.97).

#### 4.2.2 EFL teacher feedback literacy scale (EFLTFL)

Combining the teacher feedback literacy models of Carless and Winstone (2023) and Boud and Dawson (2023), Xu and Zhang (2023) constructed the 16-item EFL Teacher Feedback Literacy Scale (EFLTFL) to comprise four factors: designing feedback (DF), learning feedback (LF), building feedback consensus (BFC), and modeling feedback (MF). Six questions measure designing feedback, four questions measure learning feedback, and the remaining three questions measure building consensus and modeling feedback. Responses are scored on a 5-point scale (1 = Very inconsistent; 5 = Very compatible). Xu and Zhang (2023) achieved a Cronbach’s alpha of 0.93, whereas the current study recorded a greater internal consistency with Cronbach’s alpha of 0.90.

#### 4.2.3 Family background scale (FBC)

The Family Background Scale (FBC) measured four dimensions: Parental Education Levels (PEL), Family Income (FI), and Family Educational capital (FE). PEL was measured from 1 (Primary School) to 7 (University and above). FI was categorized from 1 (Less than ¥30,000) to 5 (More than ¥300,000). FE included six items evaluating factors like independent thinking skills, social experiences, upbringing stability, educational philosophy, parental involvement, and homeschooling resources, rated on a five-point scale. The FBC demonstrated good internal consistency with a Cronbach’s alpha of 0.85.

### 4.3 Data processing

Quantitative methods were utilized to explore the relationships between critical thinking styles, teacher feedback literacy, and family background. Descriptive statistics summarized data from the UFCTI. The chi-square test was employed to investigate the association between categorical variables, such as family income or teacher feedback literacy, and critical thinking styles. Furthermore, multivariate logistic regression analysis was utilized to assess the collective influence of various predictive factors, including family income, parents’ education level, family education capital and teacher feedback literacy, on critical thinking styles. The choice of these statistical methods was dictated by the specific nature of the research question and the unique characteristics of the data set. All analyses were conducted using SPSS, with significance set at *p* < 0.05. The value of research variables can be seen in Table 1.

**Table 1:** Research variables.

Variables	Coding
Parental Education Levels (PEL)	Low = 0 (Primary School, Primary School┋Junior High School, and above),
	Middle = 1 (Junior high school, Junior high school┋High school and above, High school)
	High = 2 (High School┋University and above, University and above)
Family Income (FI)	Low = 0 (Less than ¥60,000)
	Middle = 1 (¥60,000- ¥300,000)
	High = 2 (More than ¥300,000)
Family Educational Capital (FE)	Low = 0 (≤3), High = 1 (>3)
EFLTeacher Feedback Literacy (TFL)	Low = 0 (≤3), High = 1 (>3)
Critical Thinking Styles (CTS)	Seeker = 0, Engager = 1

## 5 Results

### 5.1 Students’ critical thinking styles

Statistical analyses were performed using SPSS 27.0 software. Overall, the students’ UFCTI scores ranged from 52.93 to 106.71 (M = 80.16, SD = 4.81). In addition, the overall seeking score was 47.75 (SD = 8.56), and the mean engagement score was 32.41 (SD = 9.33). Lamm and Irani (2011) posited that those with an overall UFCTI score of 79 or higher were identified as seekers, and those with a score of 78 or lower were identified as engaged. There are 608 Seekers (42%) and 846 Engagers (58%) (see Table 2).

**Table 2:** UFCTI score and critical thinking styles of undergraduates.

Variable	*n*	*M*	*SD*	*Min*	*Max*	Percentage
Overall UFCTI Score	1,454	80.16	4.81	52.93	106.71	
Seeker Subscale	1,454	47.75	8.56	13	65	
Engager Subscale	1,454	32.41	9.33	13.06	65.31	
CT Style: Seeker	608	84.19	4.51	52.93	78.99	42%
CT Style: Engager	846	77.26	2.25	79.05	106.71	58%

### 5.2 Influence of family background and EFL teacher feedback literacy on undergraduates’ critical thinking styles

The current study focuses on examining the impact of family background and teacher feedback literacy on undergraduates’ critical thinking styles. Using critical thinking styles (Seeker = 0, Engager = 1) as the dependent variable, chi-square tests were conducted with parental education levels, family income, family education, and teacher feedback literacy as independent variables. To control for the potential influence of sex and major, these two variables were also included as independent variables in the chi-square tests. The results showed no significant differences in the distribution of critical thinking styles across different genders (*p* = 0.216) and majors (*p* = 0.281).

The results showed that (1) the higher the parental education level, the greater the likelihood of students adopting an engager style (X^2^ = 7.647, *p* < 0.05); (2) family income exceeding 300,000 yuan significantly increased the probability of students exhibiting an engager style (X^2^ = 13.434, *p* < 0.01); (3) higher level of family education capital was associated with a greater probability of students being engagers (X^2^ = 12.641, p < 0.05) (see Table 3).

**Table 3:** The result of chi-square analysis for undergraduates’ critical thinking styles according to family background and teacher feedback literacy (*n* = 1,454).

Item	Total	Seeker		Engager		*X^2^*	*p*
		*n*	*Percent*	*n*	*Percent*		
Sex							
Male	529	210	39.70%	319	60.30%	1.533	0.216
Female	925	398	43.03%	527	57.00%		
Major							
Social science	430	179	41.63%	251	58.37%	2.538	0.281
Natural science	764	331	43.32%	433	56.68%		
Engineering	260	98	37.69%	162	62.31%		
Parental Education Levels (PEL)							
Low	379	181	47.76%	198	52.24%	7.647	0.022
Middle	902	361	40.02%	541	59.98%		
High	173	66	38.15%	107	61.85%		
Family Income (FI)							
Low	520	220	42.31%	300	57.69%	13.434	0.001
Middle	801	352	43.95%	449	56.05%		
High	133	36	27.07%	97	72.93%		
Family Educational Capital (FE)							
Low	631	297	47.07%	334	52.93%	12.641	0.000
High	823	311	37.79%	512	62.21%		
EFL Teacher Feedback Literacy (TFL)							
Low	534	180	33.71%	354	66.29%	22.802	0.000
High	920	428	46.52%	492	53.48%		

These results support hypothesis 1, indicating that family background exerts a robust influence on critical thinking style formation. Students from more privileged families are more likely to display the dialogic reasoning, proactive inquiry and argumentation confidence characteristic of engagement-based styles, whereas students from less advantaged backgrounds are more likely to be classified as information-seeking thinkers. This pattern is consistent with cultural capital and social cognitive perspectives, which emphasize the joint influence of family resources and modeled intellectual behaviors on self-efficacy and preferred reasoning approaches.

Moreover, higher EFL teacher feedback literacy was associated with a lower likelihood of students adopting an engager style (X^2^ = 22.802, *p* < 0.05) (see Table 3). These results support hypothesis 2, which suggests that higher EFL teacher feedback literacy fosters the development of seeking-related orientation rather than engaging.

### 5.3 The interaction between family background and EFL teacher feedback literacy

To further explore the interplay between family background and teacher feedback literacy, a binary logistic regression was conducted with critical thinking style (Seeker = 0, Engager = 1) as the dependent variable. The independent variables were parental education level (PEL), family income (FI), family educational capital (FE), EFL teacher feedback literacy (TFL), and a series of interaction terms: FE × FI × PEL × TFL, FI × PEL × TFL, FE × FI × TFL, PEL × TFL, FI × TFL, and FE × TFL. A backward LR method was adopted. Model 7 was retained as the final model because the Hosmer-Lemeshow test for Model 8 (x^2^ = 8.616, df = 3, p < 0.05) indicated poor model fit (see Table 4).

**Table 4:** The result of multinomial logistic regression for undergraduates’ critical thinking styles according to family background and teacher feedback literacy (*n* = 1,454).

Model	Item	*B*	*p*	*OR*	*95%CI*		Hosmer-Lemeshaw test		
							*x^2^*	*df*	*p*
Model 1	PEL	0.29	0.073	1.336	0.973	1.834	5.622	7	0.585
	FI	−0.037	0.816	0.964	0.708	1.313			
	FE	0.154	0.429	1.166	0.796	1.708			
	TFL	−0.826	0.002	0.438	0.26	0.736			
	FE by FI by PEL by TFL	0.071	0.811	1.073	0.602	1.914			
	FI by PEL by TFL	−0.052	0.868	0.949	0.515	1.751			
	FE by FI by TFL	0.196	0.588	1.216	0.599	2.47			
	PEL by TFL	−0.242	0.342	0.785	0.477	1.293			
	FI by TFL	0.051	0.884	1.052	0.532	2.079			
	FE by TFL	0.411	0.173	1.508	0.835	2.725			
	constant	0.415	0.018	1.514					
Model 2	PEL	0.288	0.074	1.333	0.972	1.829	5.615	8	0.69
	FI	−0.026	0.852	0.974	0.739	1.283			
	FE	0.152	0.434	1.164	0.796	1.703			
	TFL	−0.807	0	0.446	0.284	0.7			
	FE by FI by PEL by TFL	0.046	0.849	1.047	0.651	1.684			
	FI by PEL by TFL	−0.022	0.926	0.978	0.619	1.548			
	FE by FI by TFL	0.23	0.396	1.259	0.74	2.144			
	PEL by TFL	−0.247	0.325	0.781	0.477	1.278			
	FE by TFL	0.405	0.176	1.5	0.834	2.696			
	constant	0.41	0.017	1.507					
Model 3	PEL	0.289	0.072	1.335	0.974	1.829	5.525	8	0.7
	FI	−0.032	0.798	0.968	0.757	1.239			
	FE	0.153	0.43	1.165	0.797	1.703			
	TFL	−0.81	0	0.445	0.285	0.695			
	FE by FI by PEL by TFL	0.03	0.86	1.031	0.736	1.444			
	FE by FI by TFL	0.231	0.396	1.259	0.74	2.144			
	PEL by TFL	−0.257	0.266	0.774	0.492	1.216			
	FE by TFL	0.412	0.155	1.51	0.856	2.665			
	constant	0.413	0.015	1.511					
Model 4	PEL	0.289	0.072	1.335	0.974	1.829	5.459	8	0.708
	FI	−0.033	0.79	0.967	0.756	1.236			
	FE	0.153	0.429	1.166	0.797	1.704			
	TFL	−0.824	0	0.439	0.288	0.668			
	FE by FI by TFL	0.265	0.153	1.304	0.906	1.877			
	PEL by TFL	−0.236	0.235	0.79	0.535	1.166			
	FE by TFL	0.403	0.157	1.497	0.856	2.618			
	constant	0.413	0.015	1.512					
Model 5	PEL	0.283	0.075	1.327	0.972	1.811	2.498	6	0.869
	FE	0.147	0.445	1.158	0.795	1.687			
	TFL	−0.827	0	0.437	0.288	0.665			
	FE by FI by TFL	0.232	0.093	1.262	0.962	1.654			
	PEL by TFL	−0.232	0.242	0.793	0.538	1.169			
	FE by TFL	0.429	0.11	1.536	0.908	2.599			
	constant	0.399	0.013	1.491					
Model 6	PEL	0.299	0.057	1.349	0.991	1.836	1.213	6	0.976
	TFL	−0.869	0	0.419	0.28	0.629			
	FE by FI by TFL	0.232	0.093	1.262	0.962	1.654			
	PEL by TFL	−0.248	0.207	0.78	0.531	1.147			
	FE by TFL	0.576	0.002	1.778	1.232	2.568			
	constant	0.441	0.004	1.555					
Model 7	PEL	0.141	0.134	1.152	0.957	1.385	4.816	7	0.682
	TFL	**−1.052**	**0**	**0.349**	**0.261**	**0.468**			
	FE by FI by TFL	0.215	0.118	1.24	0.947	1.624			
	FE by TFL	**0.56**	**0.003**	**1.751**	**1.213**	**2.527**			
	constant	0.564	0	1.758					
Model 8	TFL	−1.071	0	0.343	0.256	0.459	8.616	3	0.035
	FE by FI by TFL	0.242	0.075	1.274	0.976	1.664			
	FE by TFL	0.585	0.002	1.794	1.246	2.585			
	constant	0.676	0	1.967					

PEL, Parental Educational Levels; FI, Family Income; FE, family educational capital; TFL, EFL teacher feedback literacy. In Model 7, bold values indicate statistically significant effects in this study.

**Figure 2:**
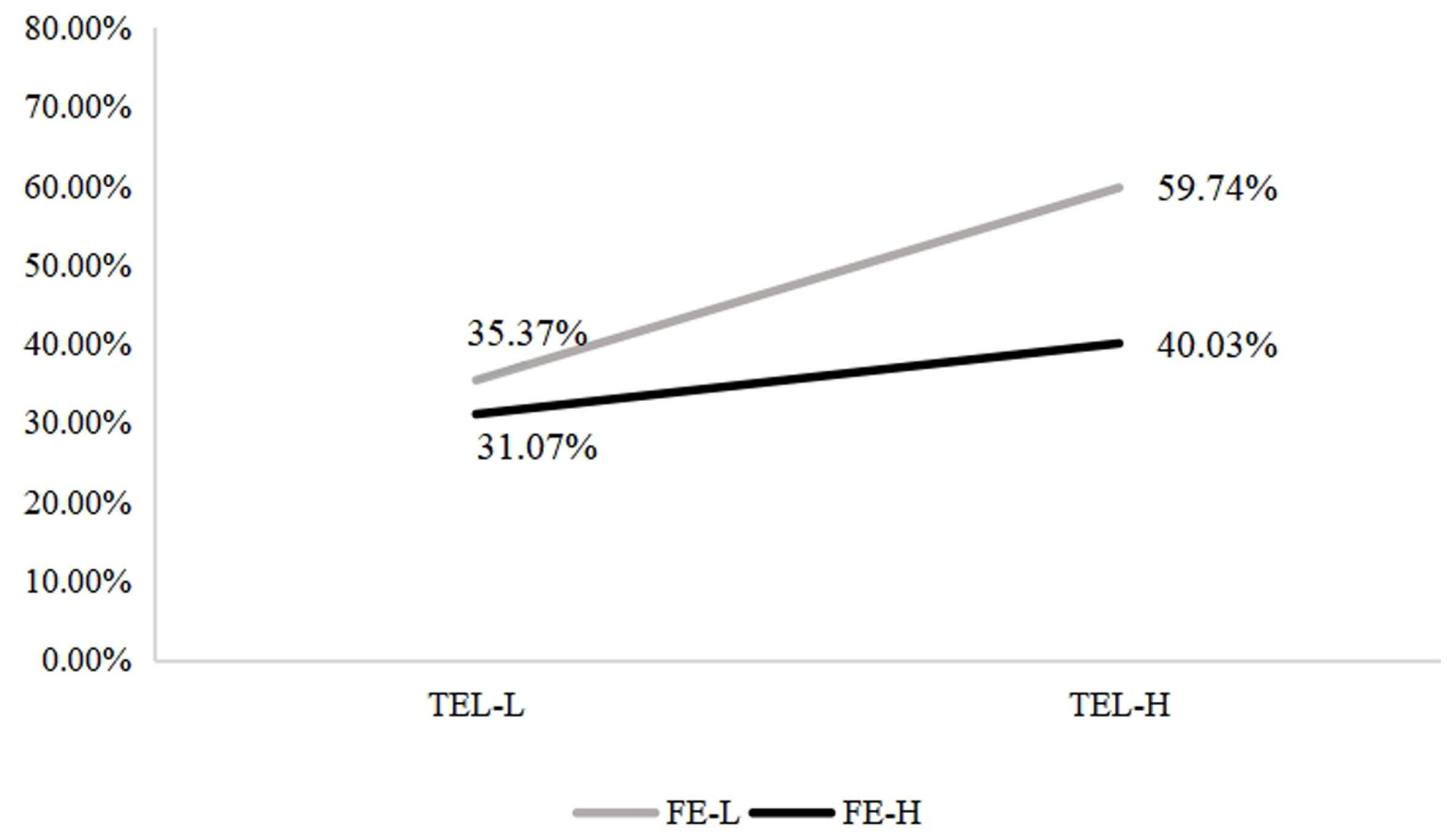
The interaction of family educational capital (FE) and EFL teacher feedback literacy (TEL) on the percentage of Seekers. FE-L = Lower level of family educational capital, FE-H=Higher level of family educational capital, TFL-L = Lower level of EFL teacher feedback literacy, TFL-H=Higher level of EFL teacher feedback literacy.

**Figure 3:**
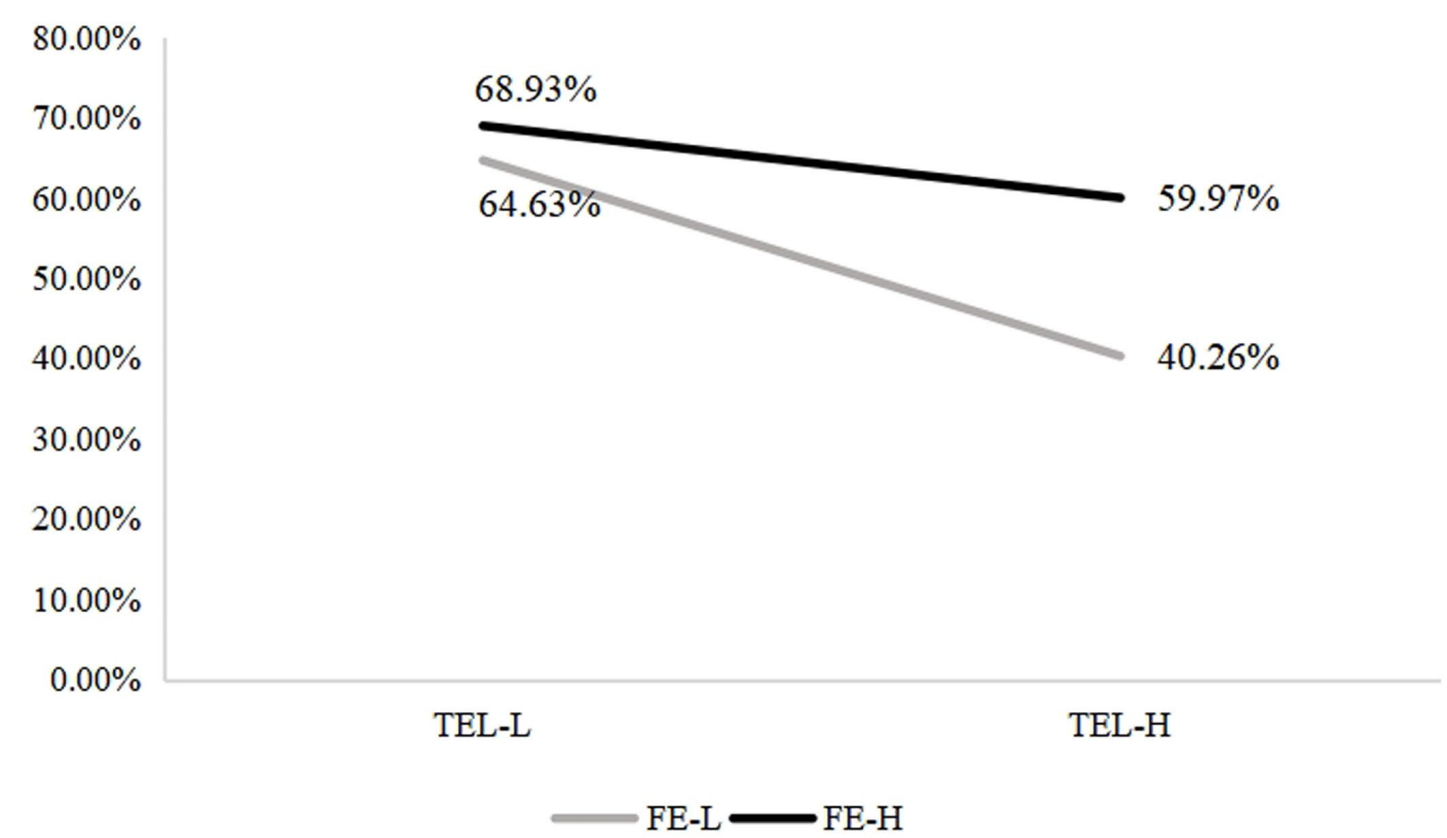
The interaction of family educational capital (FE) and EFL teacher feedback literacy (TEL) on the percentage of Engagers. FE-L = Lower level of family educational capital, FE-H=Higher level of family educational capital, TFL-L = Lower level of EFL teacher feedback literacy, TFL-H=Higher level of EFL teacher feedback literacy.

The results showed that the interaction between family educational capital (FE) and teacher feedback literacy (TFL) was significant (FE × TFL: OR = 1.751, 95% CI = 1.213–2.527), indicating that the effect of teacher feedback literacy on students’ critical-thinking styles differed across family backgrounds. When TFL was high, the proportion of students adopting a Seeker style was 40.03% among high-FE families and 59.74% among low-FE families; under lower TFL conditions the corresponding figures fell to 31.07 and 35.37%, respectively (see Figure 2). Regarding the Engager style, at high TFL the proportions were 59.97% (high-FE) vs. 40.26% (low-FE); under low TFL they increased to 68.93 and 64.63%, respectively (see Figure 3). Thus, both background groups shift toward the Seeker style when teacher feedback literacy is high and toward the Engager style when feedback literacy is low. Importantly, the upward shift toward the Seeker style under high TFL is substantially larger for students from low-FE families, indicating that teacher feedback literacy exerts a disproportionately stronger effect for these learners. These patterns suggest that the EFL teachers’ feedback literacy may alter the cognitive demands on students and thereby steer their preferred reasoning style.

Taken together, these results support hypothesis 3 and demonstrate a compensatory interaction pattern: high EFL teacher feedback literacy has a disproportionately strong impact on students from less advantaged family backgrounds, increasing their likelihood of adopting a feedback-driven, information-seeking style. In contrast, students with high family educational capital, who typically receive more cognitive modeling and reasoning support at home, show smaller shifts in thinking style under the same instructional conditions. At the same time, it is worth noting that lower teacher feedback literacy in Chinese EFL teaching practice appears to lead students from different family backgrounds to be more likely to opt for an engagement-based critical thinking style, perhaps unexpectedly. This phenomenon may stem from the fact that, when instructional design lacks feedback literacy, the tasks pose relatively low cognitive challenge to students and teacher support is insufficient. In such situations, students have no choice but to rely on peer discussion and collaboration to solve problems.

## 6 Discussion

### 6.1 The influence of family background on undergraduates’ critical thinking styles

The present study shows that all three dimensions of family background, namely parental education, family income and family educational capital, are systematically associated with undergraduates’ critical thinking (CT) styles. Students whose parents have higher educational attainment, whose families report higher income, and who possess richer educational capital at home are more likely to be classified as Engagers, characterized by dialogic, collaborative, and debate-oriented approaches to problems. By contrast, students from less advantaged backgrounds are more likely to adopt a Seeker style, marked by greater reliance on external information, structured guidance, and stepwise processing. Importantly, these results should not be interpreted as one group “thinking better” than the other; rather, they point to patterned differences in how students habitually engage with complex tasks and in the kinds of epistemic practices they find comfortable or legitimate.

This pattern is broadly consistent with recent work on CT skills and dispositions. Deng et al. (2023) showed that among Chinese primary school students, parental education and parenting styles jointly predicted both CT skills and dispositions, even after controlling for prior achievement, indicating that family-origin factors shape children’s preparedness to think critically beyond mere performance levels. In the higher education context, Yurt and Kara (2025) found that parental attitudes and school climate jointly predicted university students’ willingness to engage in critical thinking in inclusive classrooms, suggesting that family-level expectations and support continue to shape how actively students enter into critical dialogue. Taken together with our findings, this emerging evidence base suggests that the “readiness to engage” facet of CT is strongly conditioned by family-origin resources and socialization processes.

Our findings further highlight the distinctive role of family educational capital, operationalized via home learning resources, parental involvement, stability of upbringing, and educational philosophy, over and above conventional indicators such as parental education and income. This aligns with an expanding body of research on family cultural capital in East Asian contexts. Using PISA 2022 data, Djekourmane et al. (2025) showed that family cultural capital (e.g., books, learning materials, and education-related practices) directly predicts students’ cognitive performance across multiple countries and indirectly operates through academic engagement and self-efficacy. In China, Yu et al. (2022) reported that various forms of family cultural capital influence junior high students’ academic achievement partly through home reading habits and learning engagement, while Jin et al. (2024) found that institutionalized and objectified cultural capital significantly predict middle-school achievement. Our construct of family educational capital is conceptually close to these notions, but extends them by demonstrating that such capital is not only related to how well students perform, but also to the CT styles toward which they gravitate. High family educational capital appears to cultivate dispositions of confidence, conversational norms, and argument-oriented interaction that underpin an engagement-oriented style; when these resources are scarce, students may instead rely more heavily on structured, information-seeking strategies.

At a more fine-grained level, previous research has begun to unpack the micro-processes through which home experiences translate into higher-order cognition. Lurie et al. (2021) showed that socioeconomic differences in early academic achievement are mediated by the degree of cognitive stimulation in the home and children’s language development, underscoring the importance of everyday conversation, learning materials, and caregiver involvement. Von Stumm et al. (2020) similarly demonstrated that preschool verbal and non-verbal abilities, themselves socially patterned by family socioeconomic conditions, account for a substantial portion of the link between family background and later school performance. More recent neuroscientific work indicates that cognitively stimulating, language-rich environments are associated with differences in children’s functional brain architecture in networks supporting working memory and cognitive control (Rakesh et al., 2024). Although these studies do not measure CT styles directly, they collectively suggest that families with higher education, income, and educational capital are better positioned to provide cognitively rich, linguistically demanding micro-environments that foster exploratory, discussion-based reasoning. Our engager group, predominantly drawn from high-education, high-income, high-capital families, plausibly benefits from precisely these patterns of stimulation and interaction.

In sum, the study demonstrates that family background in contemporary Chinese higher education is a powerful organizer of CT style. It corroborates recent evidence that family background is a key factor in the development of critical thinking (e.g., Deng et al., 2023; Vidal et al., 2023), while extending this work by showing that it also shapes how students prefer to think, that is, whether through dialogic engagement or structured information seeking. This style-level perspective helps explain why undergraduates from less advantaged families may appear quieter, more cautious, or less verbally assertive in dialogic tasks, even while they are actively exercising critical judgment through careful information gathering and methodical processing.

### 6.2 The influence of EFL teachers’ feedback literacy on undergraduates’ critical thinking styles

Our findings indicate that EFL teacher feedback literacy (TFL) is systematically related to undergraduates’ critical thinking (CT) styles. As hypothesized, higher TFL was associated with a lower probability of students being classified as Engagers and a higher probability of being classified as Seekers. In other words, in Chinese university EFL classrooms, feedback-literate teachers appear to channel students’ critical work into a feedback-driven, information-seeking style, characterized by careful gathering, checking and applying of feedback, rather than into overt dialogic engagement. This pattern suggests that, under conditions of high TFL, CT is more likely to be enacted through text-mediated information processing than through visible, debate-oriented interaction.

This interpretation is consistent with recent conceptualizations of teacher feedback literacy as the capacity to design and orchestrate feedback processes that students can meaningfully use (Boud and Dawson, 2023; Carless and Winstone, 2023). de Kleijn et al. (2023) further characterizes student feedback processes as cycles of seeking, making sense of, using, and responding to feedback. Our data suggest that when EFL teachers are highly feedback-literate, planning staged feedback tasks, articulating criteria, and providing information-rich comments, students are nudged toward precisely these activities. The Seeker style observed in our sample can therefore be read as the cognitive “signature” of such environments: students devote substantial time and cognitive resources to consulting comments, comparing drafts with exemplars, and verifying their alignment with rubrics before committing to a position. Rather than contradicting existing feedback literacy literature, our results extend it by demonstrating that feedback-literate teaching in large-scale EFL classrooms is associated with a particular mode of CT, namely an information-seeking, feedback-mediated orientation.

Empirical work on student feedback literacy and self-regulated learning provides additional support. Woitt et al. (2025) demonstrate that feedback-literate students are distinguished by their tendency to interrogate and organize feedback information rather than passively receive it. Similarly, Yang and Zhang (2023) found that Chinese EFL writers who more actively planned, monitored, and evaluated their use of feedback reported higher self-regulation and deeper engagement with revisions. Bellhäuser et al. (2023) further demonstrate that frequent, adaptive feedback can strengthen university students’ daily self-regulated learning strategies. Taken together, these studies depict feedback-rich environments in which students’ critical work is heavily invested in processing information, managing revisions and monitoring quality, behaviors that are closely aligned with our operationalization of the Seeker style. Our findings empirically link this process-oriented feedback literature to relatively stable CT style preferences, suggesting that high TFL may “stabilize” an information-seeking orientation as students’ habitual way of engaging with academic tasks.

At the same time, our results converge with research showing that many EFL learners define “good feedback” in strongly informational terms. Large-scale studies of Chinese and other EFL university students report pronounced preferences for detailed written corrective feedback, explicit suggestions for revision, and annotated exemplars (Almanea, 2025; Almohawes, 2025). Recent classroom investigations similarly indicate that when teachers provide multi-source, rubric-based feedback (teacher, peer, and sometimes automated), students tend to invest substantial effort in reading, sorting, and integrating comments, sometimes at the expense of spontaneous oral participation (Cheng and Liu, 2022; Divekar et al., 2022; Wu et al., 2022). Our findings are broadly consistent with these patterns: high TFL appears to reinforce a mode of CT in which learners “think with feedback” through intensive information processing rather than through visible, in-class argumentation.

The association between high TFL and greater Seeker prevalence also speaks to critical perspectives on feedback literacy. Nieminen and Carless (2023) caution that feedback literacy discourse can inadvertently individualize responsibility for using feedback and obscure contextual constraints such as large class sizes, linguistic demands, and assessment pressures. In such settings, rich feedback can impose considerable cognitive load, especially on students with limited language proficiency or limited experience in working with feedback. Research on feedback shows that dense or complex feedback can increase cognitive load and reduce mindful processing unless carefully scaffolded (Berndt et al., 2022; Zhang et al., 2025). Our finding that Engager membership declines as TFL rises may reflect a similar trade-off: students withdraw from visible discussion not because their CT weakens, but because they reallocate scarce cognitive resources to decoding and applying challenging feedback. This nuance qualifies overly optimistic claims that feedback literacy will simply “unlock” engagement; instead, it may re-locate engagement from talk to text-mediated information work.

In the Chinese university EFL context, these dynamics are intensified by exam orientation and large enrolments. Prior studies have shown that College English classes often privilege accuracy, task completion, and exam preparedness, encouraging teachers to prioritize detailed written comments and criteria-referenced guidance (Lu et al., 2022; Zhang and Hyland, 2022). Under such conditions, our results suggest that high TFL is more likely to cultivate a Seeker profile, that is, students who carefully manage feedback to avoid errors and meet standards, than to produce dialogic Engagers. This does not imply that engagement-oriented critical thinking cannot be fostered; rather, it suggests that enhancing TFL alone is insufficient, and that parallel attention is needed to designing dialogic spaces, such as feedback conferences, peer review discussions and co-construction of criteria, where students can interrogate feedback, test ideas publicly and co-construct evaluative judgments.

Overall, our findings show that high EFL teacher feedback literacy tends to steer undergraduates’ critical thinking toward a feedback-driven, information-seeking style. By contrast, under lower levels of feedback literacy, students in our sample were more likely to be classified as Engagers, relying more on peer discussion and collaborative problem-solving to make sense of tasks. This contrast underscores that feedback literacy is not a neutral good, but a contextualized pedagogical resource that shapes how critical thinking is enacted, whether through dialogue, through feedback-mediated information work, or through a carefully balanced combination of the two.

### 6.3 The interaction between family background and EFL teachers’ feedback literacy

The present study identified a significant interaction between family educational capital (FE) and EFL teacher feedback literacy (TFL) in predicting undergraduates’ critical thinking (CT) styles. As TFL increased, students from both high- and low-FE families were more likely to be classified as information-seeking “Seekers,” with this shift markedly larger among students from low-FE backgrounds. Conversely, under low TFL conditions, students in both groups showed a higher probability of being classified as engagement-oriented “Engagers,” and the gap in style distributions between high- and low-FE families narrowed. This pattern directly supports H3, indicating that EFL teacher feedback literacy functions as a context-sensitive moderator of family educational capital effects on CT styles rather than as a simple equalizer.

These findings can be situated within a broader body of work on resource (in)equality. Longitudinal research in tracked education systems suggests that the interplay between individual agency and social origin may follow either a resource multiplication pattern, in which agentic resources amplify high-SES advantages, or a resource substitution or compensation pattern, in which they partly offset the constraints of low socioeconomic background on academic transitions (Mele et al., 2023). In our data, high TFL appears to operate in a compensatory manner. For students with low family educational capital, who lack dense home-based cognitive scaffolding, exposure to feedback-literate teachers, who provide criterial information, exemplars and revision-oriented comments, has a stronger style-influencing effect, steering them toward a feedback-driven, information-seeking orientation.

Recent research provides converging support for this interpretation. Ma et al. (2025) found that teacher support significantly moderated the association between family socioeconomic status and CT dispositions. More broadly, Gaddis and Murphy (2024) and Richards (2022) show that high-quality school inputs can buffer some of the disadvantages associated with low SES, especially for higher-order cognitive outcomes. Our findings extend these insights to CT styles. Rather than simply raising or lowering CT levels, TFL appears to interact with family capital to influence how students habitually engage in critical thinking, either through dialogue or through systematic information seeking. For students with low family educational capital, high TFL exerts a stronger style-influencing effect, nudging them toward an information processing orientation grounded in feedback use.

At the same time, the interaction pattern under low TFL is equally revealing. When TFL was low, both high- and low-FE students were more likely to be Engagers, and the gap between their style distributions was attenuated. One plausible explanation is that, in the absence of well-designed feedback processes, classroom tasks tend to be less cognitively complex and teacher feedback more sporadic or superficial. Under such conditions, students cannot rely on dense, criterial information from teachers and therefore turn to peer interaction, including discussion, joint problem solving and informal explanation, to make sense of tasks. Classroom studies in Chinese tertiary EFL settings show that even loosely structured, peer-led small-group talk around writing tasks can generate substantial on-task discussion and support the production of basic argumentative moves such as stating claims and providing reasons (Li et al., 2020; Li and Zhang, 2022). However, work in computer-supported collaborative learning indicates that such “busy” interaction does not automatically yield deeper critical thinking unless tasks are conceptually demanding and intentionally scaffolded through scripts, worked examples or other regulatory supports (Strauß et al., 2025; Vogel et al., 2022; Zabolotna et al., 2025).

Taken together, these findings suggest that EFL teacher feedback literacy acts as a context-sensitive moderator of family educational capital effects rather than a straightforward equalizer. On the one hand, high TFL appears to support quality information-seeking styles among low-FE students by providing the structured feedback resources they lack at home. On the other hand, it does not automatically close gaps in engagement-oriented participation; high-FE students remain more likely to combine sophisticated feedback use with dialogic, outward-facing critical thinking.

### 6.4 Implications for educational practice and policy

The findings of this study indicate that undergraduates’ critical thinking styles, whether engagement oriented or information seeking, are affected by the interplay between family educational resources and classroom feedback environments. In light of this, three implication strands emerge:

At the instructional level, EFL teachers should design feedback mechanisms that support both engagement-oriented and information-seeking modalities. For example, feedback sequences might combine structured, information-rich comments and exemplars (supporting information-seekers) with dialogic follow-up sessions (supporting engagers) that invite students to question, argue, or build on their feedback. In contexts where students from lower family educational capital are more likely to adopt an information-seeking style, teachers can scaffold their transition by providing guided “feedback-decoding” worksheets, allocating in-class time for initial processing of comments, and pacing feedback cycles so cognitive overload is minimized. Research shows that students become active agents of feedback only when supported to interpret, plan and act on comments (Carless and Winstone, 2023).

Besides, university and educational authorities should establish a sustained professional development programme for EFL teachers centred on feedback-literacy, which emphasizes the design of feedback cycles, building students’ feedback uptake processes, and modeling learner agency. Teachers should engage in peer-observation, reflective inquiry sessions and feedback-design workshops embedded in their annual training. This initiative must address the demands of large-class, exam-oriented contexts and diverse student backgrounds by providing scalable, iterative training models with regular monitoring of teacher feedback-literacy scores and student uptake behaviors. Over time, such an intervention will help feedback become a scaffold for critical thinking rather than a mere corrective instrument.

Finally, local education authorities should launch a coherent Family Educational Capital Cultivation Initiative that builds parental capacity in three areas. (1) enriching the intellectual environment at home (e.g., providing curated book kits, prompts for family discussions and reading circles); (2) strengthening educational self-efficacy and guidance for parents (e.g., workshops on supporting children’s reasoning, decision-making in learning and peer networks of parents); and (3) cultivating learning communities between families and schools (e.g., digital forums linking parents with teachers and students, and monthly dialogue sessions involving parents, students and teachers). Particular focus should be given to households with low educational capital, as indicated by factors such as the number of learning resources at home, the frequency of academic conversations within the family, and parental involvement in school-related learning events. This strategy aims to prevent structural disparities in critical thinking styles arising from insufficient educational capital within families.

## 7 Conclusion and limitation

This study examined how family background and EFL teachers’ feedback literacy jointly affect undergraduates’ critical thinking styles, three main conclusions can be drawn. First, family background, as indicated by parental education, family income and family educational capital, systematically influenced students’ critical thinking styles.

Undergraduates from more advantaged families were more likely to adopt an engagement-oriented style, whereas those from less advantaged backgrounds tended toward an information-seeking style. Second, EFL teacher feedback literacy emerged as a style-influencing factor. In the Chinese university EFL context, higher feedback literacy was associated with a higher likelihood of adopting the Seeker style. Third, the interaction between family educational capital and EFL teacher feedback literacy confirms that classroom feedback practices do not operate in a vacuum. The significant interaction effect shows that high EFL teacher feedback literacy has a stronger impact on students from lower family educational capital backgrounds, shifting them more sharply toward a feedback-driven information-seeking style, while students from high-FE families maintain comparatively higher engagement-based orientation. Together, these findings offer a new perspective on critical thinking styles by demonstrating that they are co-produced by family cultural capital and feedback-rich EFL classroom ecologies.

Several limitations should be acknowledged. First, the sample was drawn from a university in China, which may restrict the generalizability of the findings to other educational systems, disciplines or language-learning environments. Replication in different regions and institutional types, as well as in non-EFL settings, would clarify the boundary conditions of the observed patterns. Second, the study relied on self-report survey data and a cross-sectional design; as such, causal inferences about the developmental trajectories of critical thinking styles and feedback processes remain tentative. Longitudinal and mixed-methods research, combining repeated measures of critical thinking styles, observations of feedback practices, and qualitative accounts of how students work with feedback, would provide a more dynamic picture of how styles evolve over time. Third, the present study did not directly measure student feedback literacy or classroom discourse patterns. Future research could integrate measures of student feedback literacy and micro-analyses of feedback episodes to examine more precisely how teacher and student feedback literacy interact to support both engagement-oriented and information-seeking forms of critical thinking.

Despite these limitations, this study reveals how family background and the EFL teacher feedback literacy jointly influence undergraduates’ critical thinking styles. This will help educators and policymakers design educational support systems that respect stylistic diversity while mitigating background-related inequalities.

## Data Availability

The datasets presented in this study can be found in online repositories. The names of the repository/repositories and accession number(s) can be found at: the data can be accessed through Figshare, https://doi.org/10.6084/m9.figshare.27054814.
